# Tracking and Dynamic Tuning of a Wireless Powered Endoscopic Capsule †

**DOI:** 10.3390/s22186924

**Published:** 2022-09-13

**Authors:** Lucas Murliky, Gustavo Oliveira, Fernando Rangel de Sousa, Valner João Brusamarello

**Affiliations:** 1 Department of Electrical Engineering, Universidade Federal do Rio Grande do Sul, Porto Alegre 91501-970, Brazil; 2 Department of Electrical and Electronic Engineering, Universidade Federal de Santa Catarina, Florianópolis 88040-900, Brazil

**Keywords:** endoscopic capsule, wireless power transfer, WPT dynamic tracking

## Abstract

This work presents an inductive wireless power transfer system for powering an endoscopy capsule supplying energy to power electronic devices allocated inside a capsule of ≈26.1 mm × 9 mm. A receiver with three coils in quadrature with dimensions of ≈9 mm × 9 mm × 10 mm is located inside the capsule, moving freely inside a transmitter coil with 380 mm diameter through translations and revolutions. The proposed system tracks the variations of the equivalent magnetic coupling coefficient compensating misalignments between the transmitter and receiver coils. The power on the load is estimated and optimized from the transmitter, and the tracking control is performed by actuating on a capacitance in the matching network and on the voltage source frequency. The proposed system can prevent load overheating by limiting the power via adjusting of the magnitude of voltage source $V_{S}$. Experimental results with uncertainties analysis reveal that, even at low magnetic coupling coefficients *k* ranging from (1.7 × 10${}^{-3}$, 3.5 × 10${}^{-3}$), the power on the load can be held within the range of 100–130 mW. These results are achieved with any position of the capsule in the space, limited by the diameter of the transmitter coil and height of 200 mm when adjusting the series capacitance of the transmitter in the range (17.4, 19.4) pF and the frequency of the power source in the range (802.1, 809.5) kHz.

## 1. Introduction

Capsule endoscopy is a technique that employs a tiny wireless camera embedded in a small device [1,2]. During the procedure, the device is swallowed by the patient, allowing it to take photos while travelling through the digestive tract. The characteristics of the application mean that several restrictions related to the small volume of the capsule are imposed. This constraint primarily impacts the energy autonomy of the electronic circuits, as it limits the size of the batteries. Moreover, high quality images and motion control of the capsule are necessary features for improving diagnostic ability, and cannot be achieved without expending considerable extra power [3]. To overcome these issues, Wireless Power Transfer (WPT) based on low- or mid-range inductive coupling has been considered as a possible solution for powering the electronic systems and recharging the batteries [1,4,5,6].

In recent years, many applications for restricted environments have been implemented via WPT, including sealed and embedded sensors in concrete walls [7] and various devices intended for implantation in the human body [8,9,10,11,12] that are capable of carrying both power and data [13]. In addition, WPT has found applications in consumer electronics for charging the batteries of mobile devices [14] and electrical vehicles [15,16,17,18,19].

In an inductive link, the magnetic coupling coefficient *k* between the transmitting and the receiving coils is dependent on the distance. Thus, low magnetic coupling coefficients are associated with cases where the transmitter and receiver coils are relatively far each other. A dynamic *k* is associated with cases in which one or both coils can move. Moreover, the inductive link usually works via tuning to the frequency of the transmitter power source (ETX). The syntony is defined by the equivalent circuit of the coils and a capacitive matching network. The tune of the circuit guarantees the efficiency or the maximum possible power transferred (MPPT) to the load on the receiver (ERX). However, both a the dynamic magnetic coupling coefficient *k* and dynamic loading require active (dynamic) tuning in order to track changes and maximize the power delivered to the load. Active tuning [20] can be achieved by frequency sweeping [21], using switching capacitors [22], using saturable variable inductors [23], or by a combination of techniques [24,25]. None of the aforementioned works have investigated the performance of an inductive link for very low values of *k*, as featured by long distances (>radii of the coil). Furthermore, most of the works cited above present a single variable for tuning the resonant circuit, missing the possibility of attaining extra degrees of freedom for controlling the power delivered to a load. Different optimization methods can be used to track the maximization or minimization process of generic objective functions through the variation of controllable parameters [26,27,28,29]. In the present article, an output power $P_{o}$ optimization process is implemented by varying the frequency and the capacitive network simultaneously.

This work is an extension of the work presented in [30], and focuses on the estimate of *k* between coils by measuring the voltage $V_{S}$ and current $I_{S}$ in the ETX without any intervention with respect to the ERX (where the load is connected). The main innovation presented in this article lies in the optimization procedure used to track and dynamically compensate for variations in distances, and thus for the value of *k* between the coils, without increasing the volume of the ERX, especially for applications with very low magnetic coupling coefficients $\left(k\leq 1\times 10^{-2}\right)$. Thus, we present a case study of a specific inductive link: ETX coil with a diameter of ⌀=380 mm and ERX composed by three coils in quadrature (with ⌀=9 mm ×9 mm ×10 mm) for use in a commercially available endoscopic capsule of ≈26.1 mm ×9 mm [31,32]. The geometric setup of this application is characterized by a large asymmetry between the coils. Furthermore, the ETX can move over large distances, resulting very low values of *k* in the range of $\left(1.7\times 10^{-3},3.5\times 10^{-3}\right)$. The chosen frequency was centered at 805 kHz. In this case, it is only possible to maintain the power on the load and compensate for the misalignment between coils when the designed MPPT is sensitive to the range of possible variations of the actuating parameters. Thus, the presented procedure controls the MPPT by simultaneously actuating on two variables, namely, the frequency of the power source (ω) the and ETX series capacitance ($C_{1}$). In addition, the matching capacitive network is customized with an initial value of $C_{1}$ in order to allow real-time continuous maximization of power on the load. Table 1 summarizes different techniques for dynamically tuning the WPT applied to the endoscopic capsule and compares them with the method proposed in this article.

The proposed system can both compensate the inductive link and guarantee a range of power values on the load operating on the MPPT given the actual circuit and variables ω,C. The proposed approach allows the capsule to move freely, guaranteeing minimum required power on the load by adjusting the input voltage source magnitude, implying a lower SAR level. The results show the advantages of the presented approach in compensating the WPT with low values of *k*. The power on the load is maintained above the lower limit of 100 mW on a load of 47 Ω with ETX moving inside the available free space by adjusting the frequency of the power source in the range of (802.1, 809.5) kHz and the compensating capacitance in the range of (17.4, 19.4) pF. Finally, we limited the power to 130 mW by controlling the magnitude of the power source $V_{S}$ in order to avoid overheating on the load.

## 2. Multicoil Inductive Link

**Figure 1:**
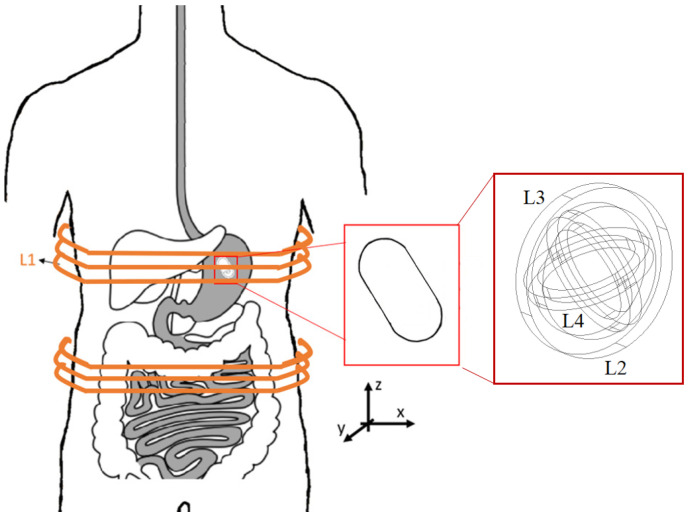
Endoscopy system showing the external coil (ETX) and detail of the capsule with four coils (L1, L2, L3 and L4) in quadrature (ERX).

The presented WPT design is asymmetrical, with a large ETX coil and a small ERX applied to the case study of a Swallowable Wireless Endoscopy Capsule (SWEC) of length × diameter ≈26.1 mm ×9 mm. The ETX is large enough to fit around the patient’s body and the ERX is small enough to fit inside the SWEC (Figure 1) with three coils in quadrature.

The ETX–ERX pairing forms a weakly coupled inductive link with a low *k* ($1.7\times 10^{-3}<k<3.5\times 10^{-3}$) because of the large distance between ETX and ERX. In clinucal application, after being swallowed the endoscopy capsule should move freely in the gastrointestinal tract while facing rotations and translation movements. The quadrature coils inside the capsule ensure minimal or critical *k* in at least one of these coils [31,35]. Thus, the receiver coils can pick up the magnetic field from three different directions and balance the output-induced voltage, providing enough power to the load in any possible position of the capsule in the gastrointestinal tract.

### 2.1. Design of the Inductive Link to the SWEC

**Figure 2:**
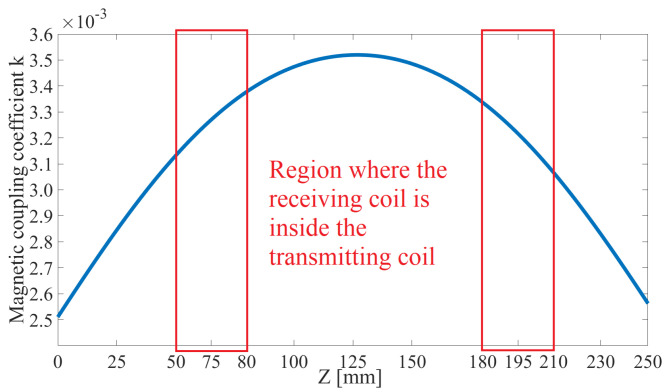
The magnetic coupling coefficient *k* between the ETX and one receiver coil aligned with the *z* axis when the ERX moves over a range of 250 mm.

**Figure 3:**
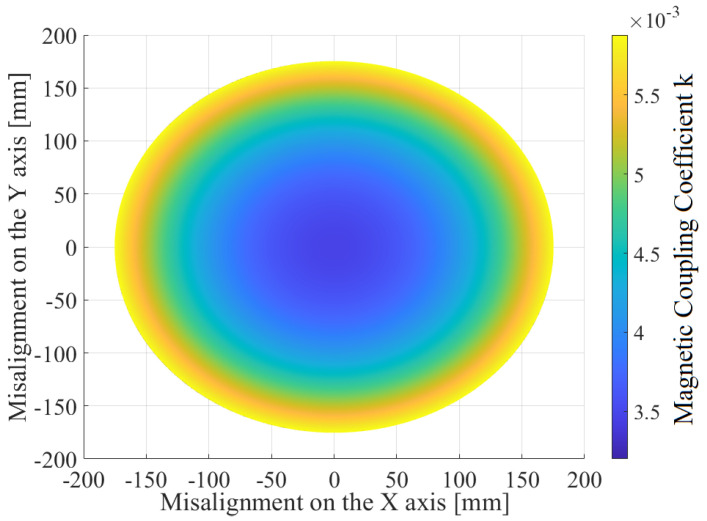
Value of *k* over xy plane when one coil of the ERX is aligned with the *z* axis at Z=125 mm (Figure 2).

Both the ETX and ERX coils are solenoids (Figure 1). The ETX coil was built with two sections, ⌀=380 mm, 43 turns, and a height of 30 mm each, presenting inductance $L_{1}$. In the capsule, three coils are mounted orthogonally; $L_{2}$ and $L_{3}$ have ⌀=9 mm with 14 turns, while $L_{4}$ has ⌀=10 mm and 14 turns. Supposing that the ETX coil is perfectly aligned with the *z* axis (Figure 1) and fully coupled to one of the receiving coils, we can disregard other receiver coils because they are in quadrature. Then, supposing that the ERX is positioned in the center of the ETX solenoid without any angular misalignment, the magnetic coupling coefficient *k* can be plotted as shown in Figure 2 when the ERX moves through the *z* axis. The red boxes depict the limits of the ETX solenoid width of 30 mm each. Figure 3 shows the values of *k* over the xy plane when ERX is aligned and centered with *z* axis at Z=125 mm.

**Figure 4:**
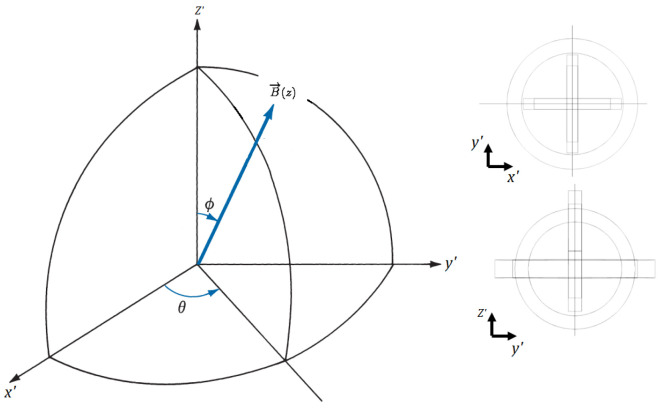
ERX Rotation angles ϕ and θ over x,y,z space (reference ETX and ERX aligned with *z* axis).

**Figure 5:**
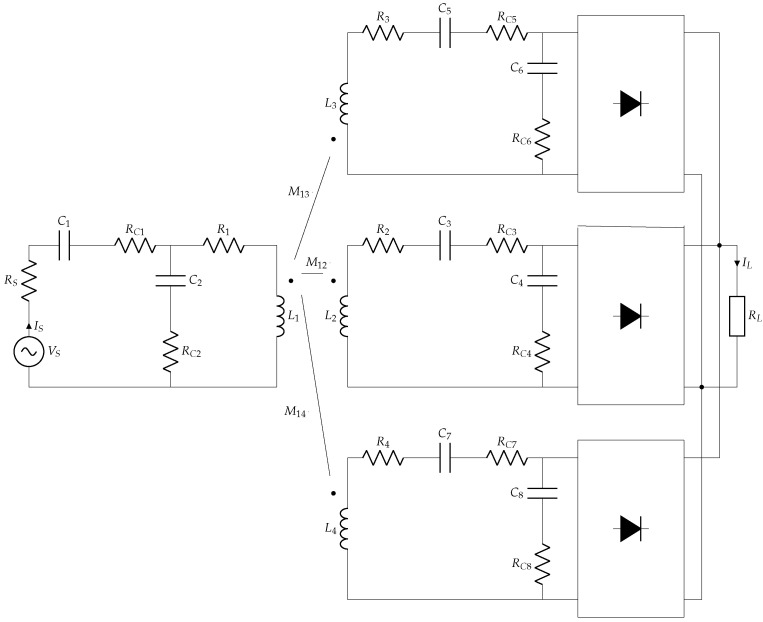
Model of the inductive link with one transmitter and three receiver coils located inside the endoscopic capsule, which moves freely through translations and rotations in the gastrointestinal tract.

As stated in [30], by rotating the capsule along the *x* and *y* axes with angles θ and ϕ (according Figure 4), the values of the mutual inductances (see Figure 5) vary as well.

Thus, three magnetic coupling coefficients between the ETX and the coils of the ERX can be described as functions of the angles θ and ϕ (1):

(1) $$\begin{array}{c} k_{1}=Ck_{0}\cos\phi \\ k_{2}=k_{0}\cos\theta \sin\phi \\ k_{3}=k_{0}\sin\theta \sin\phi \end{array}$$

where $k_{0}$ is the magnetic coupling coefficient when one of the coils from the ERX is aligned with the *z* axis and *C* is a constant describing the effects caused by differences in the diameters of the receiver coils (${⌀}_{L2}=9$ mm, ${⌀}_{L3}=9$ mm, and ${⌀}_{L4}=10$ mm, respectively; see Figure 1). Figure 4 shows the θ and ϕ angles over the adopted x,y,z space.

The mutual inductance (2) depends on the magnetic coupling coefficient *k* and on the inductances of the transmitter $L_{ETX}$ and receiver $L_{ERX}$:

(2) $$M=k\sqrt{L_{ETX}L_{ERX}}$$

The approach presented in this article is based on tracking the tuning in the transmitter circuit ETX and monitoring the effects of the reflected impedance by the ERX. Thus, the design of the coils in the ETX–ERX system must guarantee a mimimum (critical) coupling coefficient $k_{c}$ throughout the whole capsule path in order to allow the implemented circuit to detect impedance variations and track the tuning.

The designed inductive link is able to produce $k_{0}$ in the range $\left(1.7\times 10^{-3},\;3.5\times 10^{-3}\right)$ (see Figure 2 for z≈250 mm to z≈125 mm displacement and constant C≈1). In this specific case, the presented power control system can maintain the required power on the load, as far as the compensation for variations of the reflected impedance detected on the ETX during misalignments or load variations. Although the maximum power under a given pair ($\omega ,C_{1}$) is obtained when the reactance on the load is cancelled, maximum power transfer cannot be guaranteed because the resistive part of the load may not match the real part of the link impedance. Only the reactive portion of an impedance can be adjusted by varying both ω and $C_{1}$. Thus, the tuning and cancellation of the reactive portion cannot guarantee the maximum, only the “maximum possible power transfer” (MPPT) to the load.

### 2.2. Power Transfer Analysis

The multi-coil inductive link described in [30] is shown in (Figure 5). The mixed-compensation matching network is composed of four capacitors ($C_{1}-C_{4}$) in an SPSP configuration [36,37]. The ETX is powered by a voltage source $V_{S}$ with internal resistance $R_{S}$ and the ERX is connected to an AC–DC converter and to a load $R_{L}$ representing the SWEC. $R_{1}-R_{4}$ and $R_{C1}$, ..., $R_{C8}$ are intrinsic resistances of the inductances and ESR of the compensation capacitors, respectively. Finally, $M_{1N}=k_{1N}\sqrt{L_{1}L_{N}}$ are the mutual inductances, where $k_{1N}$ represents the magnetic coupling coefficient between $L_{1}$ and $L_{N}$, N=2,3,4. The cross mutual inductances of the receivers $M_{23},M_{24},M_{34}$ are disregarded because the coils are orthogonal, and thus we can assume three independent receiving coils.

**Figure 6:**
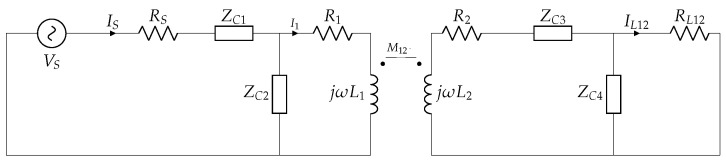
Inductive link with a single coil receiver.

Figure 6 shows part of the inductive link composed of $L_{1}$ and a single coil receiver $L_{2}$, the power source, and the compensation networks, with $Z_{Cx}=R_{x}+\frac{1}{j\omega C_{x}}$.

All circuit parameters are known except for the mutual inductance. Furthermore, the presented tracking strategy depends on the estimation of the magnetic coupling factor *k*. Thus, the voltage and the current and voltage in ETX are measured in order to infer the value of *k*. As shown in [24], the magnetic coupling factor *k* can be estimated from the measured source current ($\hat{I_{s}}$) and the circuit parameters as follows:

(3) $$\hat{k}\left(\hat{I_{s}}\right)=\pm \left|\frac{T_{1}T_{2}T_{3}}{\omega T_{4}\sqrt{L_{1}L_{2}}}\right|$$

where

$$\begin{array}{c} T_{1}=\sqrt{Rs+Z_{C1}+Z_{C2}}\\ T_{2}=\sqrt{-Vs\left(Z_{C2}+Z_{L1}\right)+\hat{I_{s}}T_{5}}\\ T_{3}=\sqrt{Z_{C4}\left(Z_{C3}+Z_{L2}\right)+R_{L12}\left(Z_{C3}+Z_{C4}+Z_{L2}\right)}\\ T_{4}=\sqrt{-\left(Rs+Z_{C1}+Z_{C2}\right)T_{6}\left(R_{L12}+Z_{C4}\right)}\\ T_{5}=\left(Z_{C2}Z_{L1}+R_{s}\left(Z_{C2}+Z_{L1}\right)+Z_{C1}\left(Z_{C2}+Z_{L1}\right)\right)\\ T_{6}=\left(-Vs+\hat{I_{s}}\left(Rs+Z_{C1}+Z_{C2}\right)\right). \end{array}$$

Thus, with the estimated value of the magnetic coupling coefficient $\hat{k}$ we can estimate the mutual inductance as defined in (2). In addition, we can describe the power on the load $R_{L12}$ as $P_{o12}=\frac{1}{2}{\left|\hat{I_{L12}}\left(\hat{I_{s}}\right)\right|}^{2}R_{L12}$, where $\hat{I_{L12}}\left(\hat{I_{s}}\right)$ is the estimate of the current on $R_{L12}$. However, as ERX is composed by three orthogonal coils, we can generalize this conclusion to the inductive links formed by $L_{1}$, $L_{3}$ and by $L_{1}$, $L_{4}$. Thus, the three inductive links are connected to rectifiers in parallel with the load [38,39] and the power on the load $R_{L}$, and the efficiency can be calculated with

(4) $$P_{o}=\frac{\max{\left(V_{L12},V_{L13},V_{L14}\right)}^{2}}{R_{L}}$$

(5) $$\eta =\frac{P_{o}}{\frac{1}{2}\;\cdot \;\mathbb{R}e\left\{\hat{V_{S}}\;\cdot \;\hat{I_{S}^{*}}\right\}}.$$

## 3. Methodology

**Figure 7:**
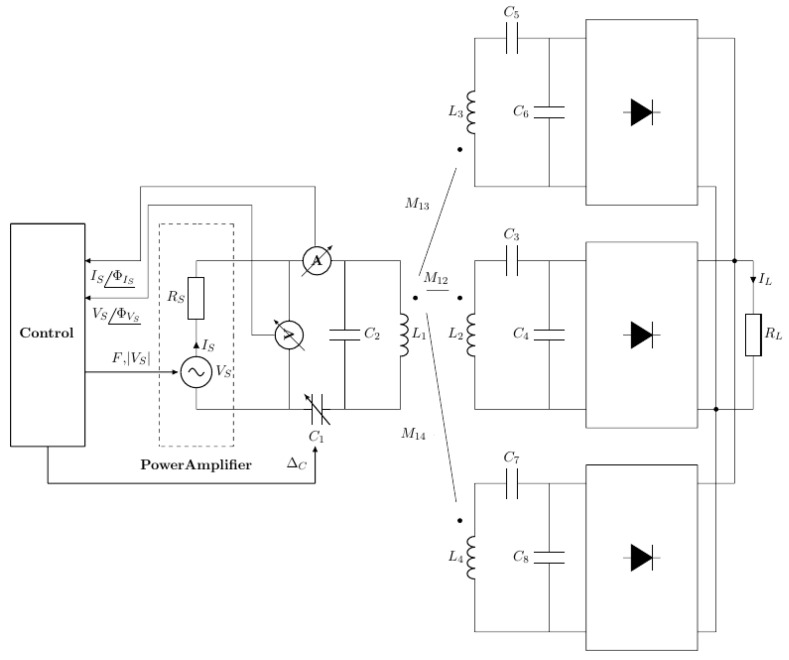
System architecture of continuous system tuning whenever the load or the relative position of the coils changes.

The estimated input current and voltage $\hat{I_{S}}$ and $\hat{V_{S}}$ are real-time monitored (Figure 7) and the control system runs an optimizing algorithm in order to maximize the power on the load by adjusting both the voltage source frequency ω and the capacitance $C_{1}$ of the ETX.

We can estimate the magnetic coupling coefficient *K* (with (3), an equivalent mutual inductance $M_{eq}=f\left(M_{12},M_{13},M_{14}\right)$) [24], and the the reflected impedance of the ETX. Thus, the control system is able to track the MPPT by running the optimization process and searching ω and $C_{1}$, reestablishing the tuning of the circuit:

(6) $$\begin{array}{cccc} \; & \underset{\omega \in R;C_{1}\in S}{\mathrm{maximize}}\; & \; & P_{o}\left(\omega ,C_{1},\hat{I_{S}}\right)=\frac{1}{2}\sum_{n=2}^{4}{\left|\hat{I_{L1n}}\left(\omega ,C_{1},\hat{I_{S}}\right)\right|}^{2}R_{L}\\ \; & \mathrm{subject}\;\mathrm{to}\; & \; & P_{o}\leq P_{max}\;mW \end{array}$$

where $\hat{I_{L1n}}$ are the electric currents supplied to the load by each coil in the ERX. In addition, a threshold for the dissipated power can be included in (6), limiting the MPPT to $P_{max}$ mW, and $P_{max}$ can be controlled by the AC power source magnitude of the ETX. The optimization process can be executed using dedicated firmware for the ETX. The proposed optimization method is better detailed in [24].

The system continuously reads $\hat{V_{S}}$ and $\hat{I_{S}}$ and estimates variations in the equivalent mutual inductance $M_{eq}=f\left(M_{12},M_{13},M_{14}\right)$ caused by variations of the magnetic coupling coefficients $k_{1n}$ and load when the capsule moves or the equivalent load changes. The proposed control architecture detects these variations and continues to track the tuning by finding a new frequency ω and new capacitance $C_{1}$ in the transmitter.

**Figure 8:**
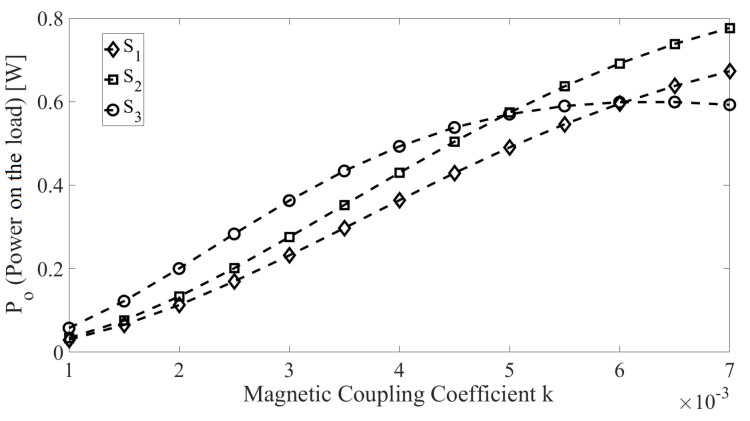
Three different sets of capacitors $\left\{S_{1},S_{2},S_{3}\right\}$ with output $P_{o}\times k$ in the range $\left(1.0\;\times \;10^{-3},7.0\times 10^{-3}\right)$ during the tuning procedure while optimizing ω and $C_{1}$; $S_{1}$ shows low performance at high values of *k*, while $S_{2}$ shows the best performance for $k>4.7\times 10^{-3}$ and $S_{3}$ for $k<4.7\times 10^{-3}$.

The *k* of the presented WPT is in the range $\left(1.7\times 10^{-3},6.5\times 10^{-3}\right)$ (Figure 3). In order to find the best matching network for this range, we applied the method presented in [36] to find 1000 sets of four compensation capacitors (fixed capacitance of $C_{2},C_{3},C_{4}$ and initial capacitance of $C_{1}$) for different values of *k*. Although these sets produce similar outputs for a specific *k*, they present different responses for the realized dynamic tuning when changing ω and $C_{1}$ during the variation of *k*. Figure 8 shows the results of three different sets of capacitors; it can be observed that the best choice is dependent on *k*.

## 4. Results

In order to evaluate the proposed method while taking into account previously mentioned restrictions in terms of dimensions, we designed an inductive link. The restrictions define the inductive link; its inductances $L_{1}$ and $L_{2}$ and respective intrinsic resistances $R_{1}$ and $R_{2}$ depend on the geometry of the coil structure, including the coil shape, copper wire thickness, wire spacing, coil size, number of turns, etc. Table 2 presents the measured parameters of the inductive link (measured with an Agilent U1730C Series LCR meter) and their respective estimated (standard) uncertainties u(x). A Rigol DG1022 function generator combined with a class-B power amplifier were used as the sinusoidal voltage source of the inductive link. Assuming fixed magnitude and frequency, the standard uncertainties can be represented by 12.0±0.1 V and $805\pm 5\times 10^{-2}$ kHz, respectively (Table 2). However, this application scans both the magnitude and frequency, and consequently the uncertainties vary with the movement of the capsule.

Measurement of the magnitude and phase of the current $\hat{I_{s}}$ were implemented with a gain and phase detector (AD8302) circuit, as presented in [40]. This IC presents a typical nonlinear gain measurement <0.5 dB in the 1.8 V range and sensitivity of 30 mV/dB. The phase measurement has a sensitivity of 10 $\mathrm{mV}{/}^{\circ }$ in the 1.8 V range. The uncertainty of the current magnitude is estimated by combining the different sources of circuit variability as $u(I_{S})\approx 8.5$ mA, assuming a specific current $I_{S}=500$ mA. As the uncertainty $u(I_{S})$ is dependent on certain variable parameters of the circuit, it can be expected to vary during compensation and maximum possible power tracking in the load. Here, the uncertainty of the measured phase was estimated as $u\left(∠I_{s}\right)\approx 0.6^{\circ }$.

The parameters and their respective uncertainties were substituted in Equations (5), (6), and (8), and the values of the magnetic coefficient *k*, power on the load, and efficiency were estimated using the Monte Carlo method [41] with 500,000 cycles (*N* = 500,000). The results for *k*, $P_{o}$, and η were obtained with a 95% confidence level, as follows: $k=\left(3.5\pm 0.2\right)\times 10^{-3}$, $P_{o}=250\pm 21$mW, and η = (7.4±0.3)% for this specific point of $I_{S}$.

**Figure 9:**
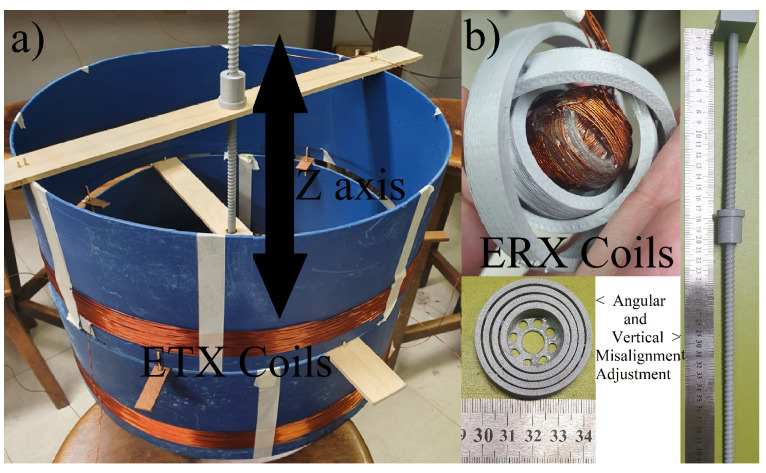
Experimental apparatus: (**a**) ETX coils with a spindle to adjust the movement of ERX on the *z* axis. (**b**) ERX coils inside a structure for varying angles ϕ and θ.

The coils were fixed and had the parameters described in Table 2. The spindle was moved to perform different values of *k* (according Figure 2) and the proposed closed-loop control system was implemented in order to track the MPPT on the load. At each movement of the vertical spindle, the coils misaligned in the *z* direction and the magnetic coupling coefficient varied as expected. The angular misalignment was implemented by placing the ERX in a movable frame to perform θ and ϕ variations according to Figure 4. The experimental coils of the ETX and ERX as well as the apparatus used to induce movement along the *z* axis (spindle) and to vary the θ and ϕ angles are shown in Figure 9. At each step, the system detected the unbalanced conditions through measurement of $V_{S}$ and $I_{S}$, actuating on ω and $C_{1}$, and re-establishing the MPPT on the load, as depicted by Figure 7.

**Figure 10:**
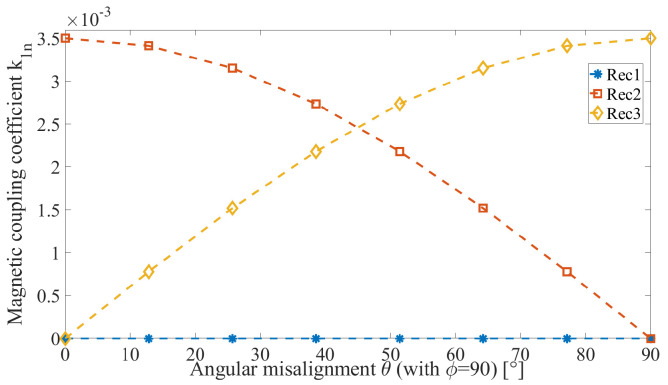
Magnetic coupling coefficient of each receiver coil while θ is varying with fixed $\phi =90^{\circ }$.

Considering the ETX coils aligned with one of ERX coils in axis *z*, depicted in Figure 4 with the ERX fixed at the spindle in the center of $L_{1}$), Figure 10 shows the variation of the magnetic coupling coefficient $k_{1n}$ while holding $\phi =90^{\circ }$ and varying θ. Rec2 illustrates the variation of the magnetic coupling coefficient $k_{13}$ between the ETX and ERX coil $L_{3}$ with ⌀=9 mm. Initially, it is completely aligned; as the angle θ increases, $k_{13}$ decreases to zero. Rec3 illustrates the variation of the magnetic coupling coefficient $k_{14}$ between the ETX and ERX coil $L_{3}$ with ⌀=9 mm, starting from zero coupling (because the initial position is orthogonal to the ETX coil), increasing the magnetic coupling as angle θ increases, and reaching the maximum at $\theta =90^{\circ }$. Rec1 represents the magnetic coupling coefficient $k_{12}$ between ETX and ERX coil $L_{2}$ with ⌀=10 mm. The magnetic coupling coefficient $k_{12}$ remains at zero because this coil remains in an orthogonal position in relation to ETX during the variation of θ. These results show that the magnetic coupling coefficients can be estimated by monitoring the effects on the ETX when only one coil is fully coupled to the transmitting system and the other two coils are in quadrature. Otherwise, there is only one measurable effect in the ETX caused by two or three magnetic coupling coefficients from different circuits.

This particular case was simulated with the Finite Element Method (FEM) using COMSOL Multiphysics software, resulting in $k_{0}=3.5\times 10^{-3}$, which satisfies Equation (1). The implemented system can estimate the equivalent magnetic coupling coefficient $k_{eq}$ from three misaligned coils. The special case of only one of the receiver coils aligned with the ETX coil produces $k_{eq}$ equal to *k* from a single coil.

**Figure 11:**
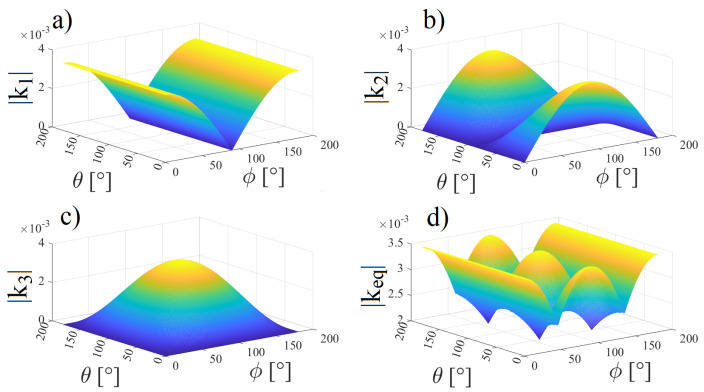
Magnetic coupling coefficients from each of the receiver coils while θ and ϕ change: (**a**) $k_{12}$, (**b**) $k_{13}$, (**c**) $k_{14}$, (**d**) combination of $k_{12}$, $k_{13}$, and $k_{14}$.

Figure 11a–c show the magnetic coupling coefficient from each of the ERX coils when the capsule is fixed at z=125 mm (see Figure 2) during the variation of angles θ and ϕ (again, the ETX and ERX ⌀=9 mm coils are aligned with the *z* axis). Figure 11d shows the combination of $k_{12}$, $k_{13}$, and $k_{14}$ while the θ and ϕ angles vary according to Figure 4.

**Figure 12:**
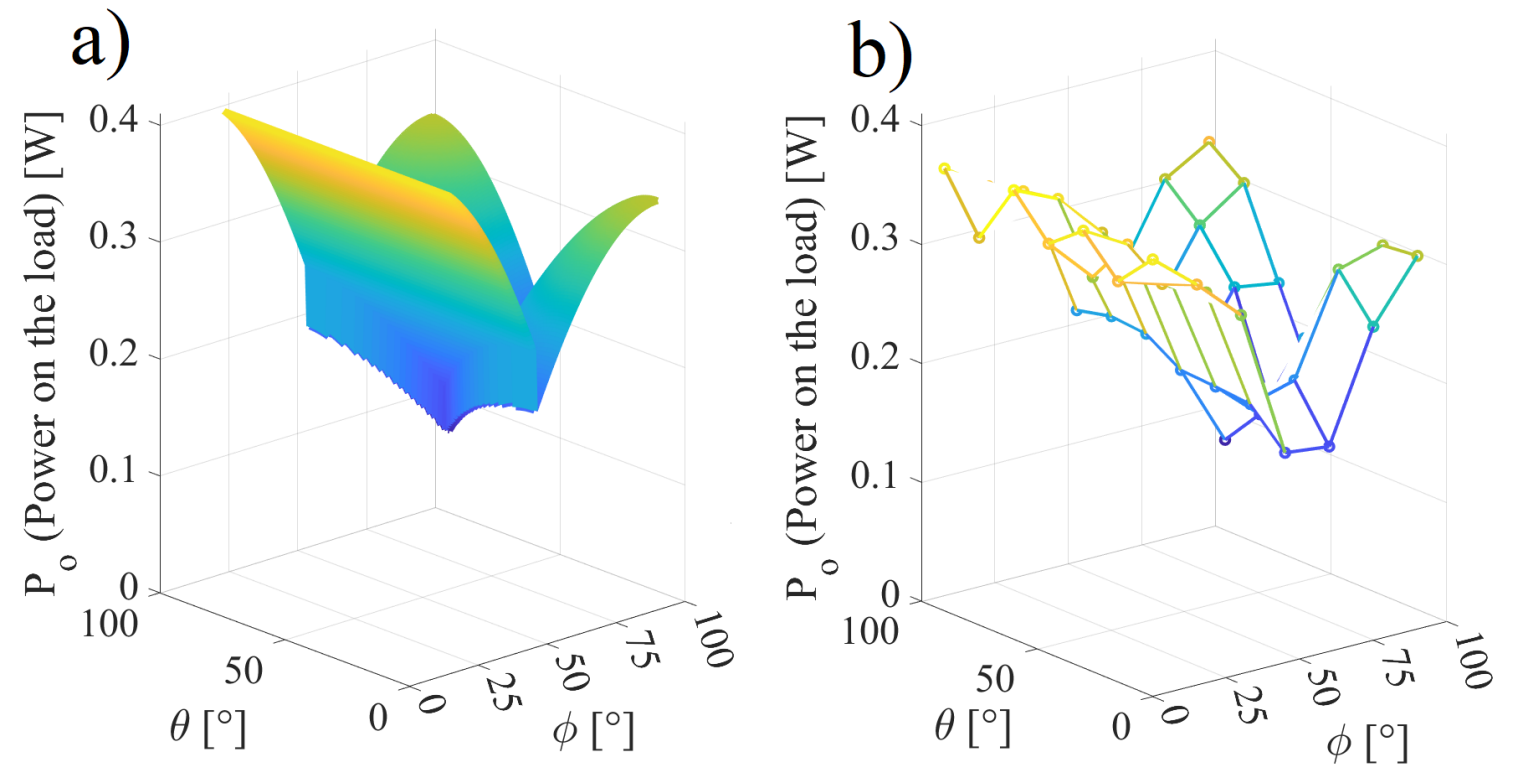
Power on the load when ω and $C_{1}$ are used to tune the inductive link: (**a**) simulated results and (**b**) experimental results.

Figure 12a,b present the respective simulated and experimental MPPT on the load with the angles θ and ϕ varying in the interval $\left(0,\;90^{\circ }\right)$. Figure 12b shows the results of 49 measured points in steps of $15^{\circ }$ of both ϕ and θ repeated three times. referring to Figure 12b, Table 3 shows points of the efficiency and power on the load with varying θ and ϕ. It can be observed that the frequency and the capacitance $C_{1}$ vary within the intervals $\left(791.3,818.0\right)$ kHz and $\left(17.69,19.10\right)$ pF for the simulated results and (802.1,809.5) kHz and (17.4,19.4) pF for the experimental results; in addition, there is a correlation of *k* with $P_{o}$ and η. Referring to Figure 12a, the mean squared error (MSE) can be calculated as MSE = 51.9 mW.

**Figure 13:**
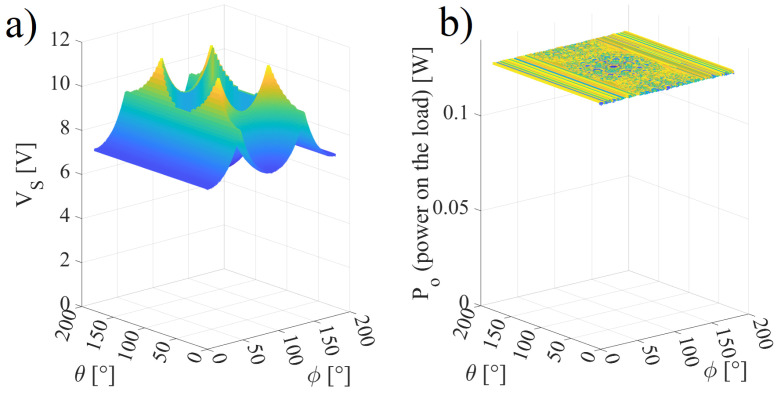
Simulated voltage $V_{S}$ with varying θ and ϕ angles and $P_{o}$ maintained at 130 mW. (**a**) Simulated Voltage; (**b**) Power on the load.

In our experiments, a power limitation was implemented in order to avoid overheating on the load. Thus, the magnitude of the voltage source voltage was controlled after the inductive link was tuned. Every time $P_{o}$ exceeded a threshold (130 mW in this work), a new magnitude of $V_{S}$ was estimated and adjusted. Figure 13a shows the variations of the magnitude of $V_{S}$ in the ETX used to order to keep $P_{o}$ limited at 130 mW in the ERX under rotation of angles θ and ϕ (Figure 13b). Table 4 presents simulated and experimental points in reference to Figure 13.

The results presented in Figure 13b are similar to the results presented in Figure 12b, with the exception that the limiting threshold of 130 mW maintains the power constant even with varying $k_{1n}$.

**Figure 14:**
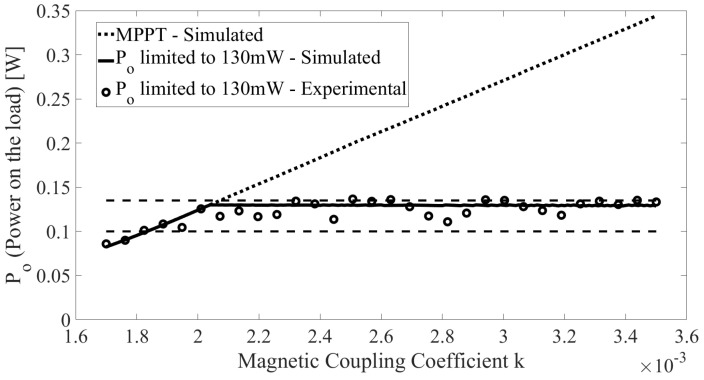
Simulated and experimental power delivered to the load after system tuning and when controlling the voltage source $V_{S}$ to avoid overheating on the load, with varying *k*.

Figure 14 shows the simulated and experimental power on the load $P_{o}$ when varying the magnetic coupling coefficient $k_{eq}$. When detecting that the ERX has moved, thereby changing the value of $k_{eq}$, ω and $C_{1}$ are adjusted to track the tuning of the circuit. The dashed lines show the limits of the interval $\left(100,130\right)$ mW. The dotted line indicates $P_{o}$ without any threshold and the solid line indicates $P_{o}$ with the threshold set at 130 mW, resulting in an MSE of =10.6 mW when considering the solid line in Figure 14 as a reference.

**Figure 15:**
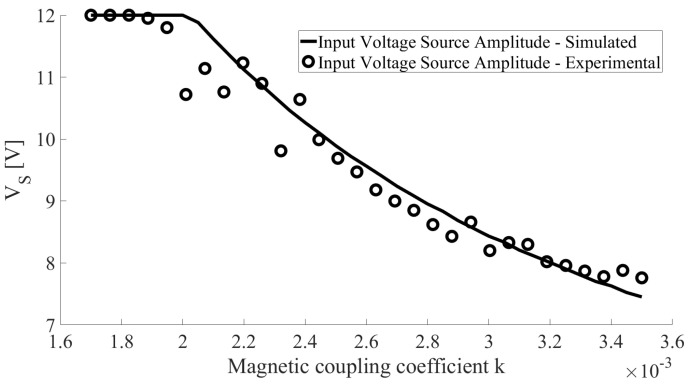
Simulated and experimental variations of the voltage magnitude $V_{S}$, limiting the power delivered to the load (130 mW in this case study) when the ERX is aligned in the center of the ETX and moving in the *z* axis, with varying *k* according Figure 2.

Figure 15 presents the simulated and experimental variations of the voltage magnitude $V_{S}$ in order to limit the power delivered to the load (130 mW in this case study) when the SWEC is aligned with the center of the ETX and moving in the *z* axis in the $\left(25,125\right)$ mm range (see Figure 2). Each position on the *z* axis presents a scaling of the variation curve of $V_{S}$ for the plane θ×ϕ, as shown in Figure 13. The experiment in Figure 15 shows 30 positions and their respective *k* values when the ERX moves along the *z* axis. This experiment was repeated three times with MSE =0.32 V, considering the simulation (solid line) as a reference.

## 5. Discussion

Table 5 shows a comparative summary of articles that have addressed the problem of WPT with the endoscopy procedure. The TX/RX column shows the *⌀* of the ETX and ERX coils, *d* is the distance between coils on the ETX (when such a structure is present), $P_{o}$ is the power on the load (mW), and η is the efficiency of the inductive link. Table 5 shows that the dimensions of the coils are somewhat similar. However, there are differences in coil structure and coil size, and several articles do not implement dynamic compensation. Consequently, there are differences in the performance of each ETX–ERX pair. In [33], the authors present an ETX with two coils, one on the floor concatenated with another coil around the patient. The authors of [42] present the implementation of a mobile capsule without any dynamic tuning, while in [3] the authors propose a modified Helmholtz transmitter coil on the ETX with dynamic tuning by switching capacitors (as in [43]). The authors of [44] present optimization of the coil geometry of the inductive link in order to maximize efficiency, and [35] present a comparison of different types of ETX and ERX coils. The authors of [31] propose an inductive link with a pair of planar transmitter coils with dynamic tuning by frequency scanning. It is notable that proposal in the present work has smaller dimensions than [3,33,35,42,43]; this parameter is important for implementation of the SWEC and has strong impact on *k*. Furthermore, the efficiency of the proposal in the present work is higher than in the other methods presented in Table 5.

The experimental results presented in this work emulate all possible movements of a capsule within the dimensions of a human torso limited by a diameter of 380 mm and a height of h=200 mm. Furthermore, the experimental results were obtained in an environment different from the final application of the capsule in the human body. Studies [44,45] have shown that the use of biological tissues causes a slight decrease in *k* and the power on the load. In this case, the same system presented in this article could be implemented, with the only difference being that the magnitude of the voltage source would have to be increased in order to maintain the load power requirements. We intend to carry out such experiments with biological tissues and SARS analysis in our future work.

## 6. Conclusions

This article presents the design, analysis, and experimental results of an IWPT system to power a capsule (≈26.1 mm × 9 mm) along a path, such as the gastrointestinal tract in an endoscopy exam. The capsule contains an ERX receiver with three solenoid-type coils (≈9 mm ×9 mm ×10 mm) moving freely through rotations and translations. The ETX transmitter is composed of two solenoids with ⌀=380 mm and width 30 mm connected in series and physically arranged concentrically at a distance of 130 mm in order to produce an approximately homogeneous internal magnetic field. As the ERX coils move in relation to the ETX coils, the magnetic coupling coefficient *k* between the emitter and the receivers varies from 0 to 1, and therefore the three coils are in quadrature, ensuring that at least one coil can pick up enough energy to power the capsule. In addition, the ERX is inaccessible, and both the adjustments and the actuation of any control action is performed in the ETX.

Thus, this work presents a procedure for continuously maximizing the power on the load by compensating for the variations caused by the movements of the coils in the ERX. The procedure is performed by measuring the voltage $V_{S}$ and current $I_{S}$ in the transmitter and simultaneously varying a capacitor in the matching network and the frequency of the AC power source in the ETX circuit. The approach of actuating on two variables instead of one has been shown to have advantages when dealing with very low magnetic coupling coefficients, such as in the case of this application ($k\approx 2\times 10^{-3}$). In addition, the procedure estimates the power on the load, checking for an upper limit and actuating on the magnitude of the voltage source $V_{S}$ in ETX (if needed), thereby avoiding overheating on the load.

The experimental results were obtained considering a minimum and maximum power at the load within in the $\left(100,130\right)$ mW range. The experiments included linear motion along an axis of ≈200 mm and two rotation angles of ERX coils. The power in the load was maintained within the proposed range by moving the ERX in the space limited by the diameter of the ETX coils and a height of up to 160 mm. In the presented case study, the variation of the magnetic coupling coefficient *k* varied within the range $\left(1.7\times 10^{-3},3.5\times 10^{-3}\right)$, which required the frequency to be adjusted in the (802.1,809.5) kHz range and the capacitance $C_{1}$ in the (17.4,19.4) pF range. Compared to similar published works found in the literature, the proposal in this work achieved higher power transfer efficiency.

## Figures and Tables

**Table 1:** Tuning methods applied to inductive links for endoscopic capsule.

Reference	Output	Dynamic Tuning Method
[31]	η	Frequency scanning
[3]	η	Capacitor switching network
[33]	η	Multiple frequency transmitter
[34]	$P_{o}$	No dynamic tuning
This work	$P_{o}$	Frequency scanning and capacitor switching network

*η* = Efficiency; *P*_o_ = Power on load.

**Table 2:** Design parameters of the inductive link.

Symbol	Parameter	Value	u(x)
*f*	Frequency	805 kHz	$5\times 10^{-2}$ kHz
$R_{S}$	Internal Resist. of $V_{S}$	700 mΩ	7 mΩ
$V_{S}$	Magnitude of $V_{S}$	12.0 V	0.1 V
$R_{1}$	ETX DC Resistance	10.18 Ω	0.05 Ω
$R_{2}$	ERX DC Resistance	572 mΩ	6 mΩ
$R_{3}$	ERX DC Resistance	588 mΩ	6 mΩ
$R_{4}$	ERX DC Resistance	603 mΩ	6 mΩ
$C_{1}$	Capacitance	18.3 p F	0.3 pF
$C_{2}$	Capacitance	2.57 p F	0.04 pF
$C_{3}=C_{5}$	Capacitance	10.22 n F	0.05 nF
$C_{7}$	Capacitance	7.22 n F	0.04 nF
$C_{4}=C_{6}=C_{8}$	Capacitance	27.5 n F	0.02 nF
$L_{1}$	ETX Inductance	1.87 mH	0.09 mH
$L_{2}$	ERX Inductance	5.04 μ H	0.08 μH
$L_{3}$	ERX Inductance	5.05 μ H	0.08 μH
$L_{4}$	ERX Inductance	7.06 μ H	0.11 μH
$Z_{L}=R_{L}$	Load Impedance	47.0 Ω	0.2 Ω

**Table 3:** Experimental and simulated data of the inductive link when the ERX coils are in the center of the ETX coils and ϕ=θ.

Simulated							Experimental			
ϕ	θ	**Freq. [kHz]**	$C_{1}$ **[pF]**	k	$P_{o}$ **[mW]**	η **[%]**	**Freq. [kHz]**	$C_{1}$ **[pF]**	k	$P_{o}$ **[W]**
0	0	791.3	19.10	$3.5\times 10^{-3}$	427.3	11.51	808.6	18.3	$\left(3.6\pm 0.2\right)\times 10^{-3}$	0.37±0.03
15	15	791.7	19.08	$3.3\times 10^{-3}$	406.4	10.50	808.5	18.3	$\left(3.2\pm 0.2\right)\times 10^{-3}$	0.33±0.03
30	30	791.7	19.08	$3.0\times 10^{-3}$	346.8	8.86	807.2	17.4	$\left(2.8\pm 0.1\right)\times 10^{-3}$	0.30±0.03
45	45	791.7	19.08	$2.4\times 10^{-3}$	252.0	6.13	809.5	17.4	$\left(2.6\pm 0.1\right)\times 10^{-3}$	0.21±0.02
60	60	818.0	17.71	$2.6\times 10^{-3}$	224.0	5.53	809.5	17.4	$\left(2.6\pm 0.1\right)\times 10^{-3}$	0.19±0.02
75	75	817.8	17.69	$3.2\times 10^{-3}$	316.2	8.13	802.1	19.4	$\left(3.4\pm 0.2\right)\times 10^{-3}$	0.29±0.02
90	90	818.0	17.71	$3.5\times 10^{-3}$	350.2	9.10	802.1	19.4	$\left(3.5\pm 0.2\right)\times 10^{-3}$	0.34±0.03

Confidence Level of *P_o_* and *k*, *CL* = 95%.

**Table 4:** Experimental and simulated data when the PDL is limited to 130 mW.

		Simulated		Experimental	
ϕ	θ	$V_{S}$	$P_{o}$ **[mW]**	$V_{S}$	$P_{o}$ **[W]**
0	0	7.28	128.7	7.8	0.13±0.01
15	15	7.49	129.6	7.9	0.13±0.01
30	30	8.14	129.6	8.0	0.14±0.01
45	45	9.61	129.5	10.0	0.13±0.01
60	60	8.17	129.6	9.5	0.13±0.01
75	75	6.95	129.8	8.0	0.12±0.01
90	90	6.59	128.9	7.5	0.13±0.01

Confidence Level of *P_o_*, *CL* = 95%.

**Table 5:** Comparative summary of results from the current literature employing WPT for endoscopy procedures.

Ref.	Freq. [kHz]	TX/RX [mm]	d [mm]	P${}_{o}$ [mW]	η [%]
[33]	1356	480/11	−	24	3.04
[42]	218	500/14	−	600	2
[3]	246	400/11	150	534	4.93
[44]	16,470	220/9	−	26	0.02
[43]	250	350/11.5	70	758	8.21
[35]	246	360/12.7	130	238.52	3.26
[31]	1000	202/8.9	100	70	1
TW	802–808	380/10	100	367.8	9.1

TW = This work.

## Data Availability

Not applicable.
